# CXCR5^+^PD1^+^ICOS^+^ Circulating T Follicular Helpers Are Associated With *de novo* Donor-Specific Antibodies After Renal Transplantation

**DOI:** 10.3389/fimmu.2019.02071

**Published:** 2019-09-10

**Authors:** Richard Danger, Mélanie Chesneau, Florent Delbos, Sabine Le Bot, Clarisse Kerleau, Alexis Chenouard, Simon Ville, Nicolas Degauque, Sophie Conchon, Anne Cesbron, Magali Giral, Sophie Brouard

**Affiliations:** ^1^Centre de Recherche en Transplantation et Immunologie UMR1064, INSERM, Université de Nantes, Nantes, France; ^2^Institut de Transplantation Urologie Néphrologie (ITUN), CHU Nantes, Nantes, France; ^3^Laboratoire Histocompatibilité et Immunogénétique - Etablissement Français du sang, Nantes, France; ^4^Centre d'Investigation Clinique en Biothérapie, Centre de Ressources Biologiques (CRB), Labex IGO, Nantes, France

**Keywords:** renal transplantation, donor-specific antibodies, DSA, circulating T follicular helper lymphocytes, Tfh

## Abstract

Donor-specific anti-HLA antibodies (DSAs) are a major risk factor associated with renal allograft outcomes. As a trigger of B cell antibody production, T follicular helper cells (Tfhs) promote DSA appearance. Herein, we evaluated whether circulating Tfhs (cTfhs) are associated with the genesis of antibody-mediated rejection. We measured cTfh levels on the day of transplantation and 1 year after transplantation in blood from a prospective cohort of 237 renal transplantation patients without DSA during the first year post-transplantation. Total cTfhs were characterized as CD4^+^CD45RA^−^CXCR5^+^, and the three following subsets of activated cTfh were analyzed: CXCR5^+^PD1^+^, CXCR5^+^PD1^+^ICOS^+^, an CXCR5^+^PD1^+^CXCR3^−^. Immunizing events (previous blood transfusion and/or pregnancy) and the presence of class II anti-HLA antibodies were associated with increased frequencies of activated CXCR5^+^PD1^+^, CXCR5^+^PD1^+^ICOS^+^, and CXCR5^+^PD1^+^CXCR3^−^ cTfh subsets. In addition, ATG-depleting induction and calcineurin inhibitor treatments were associated with a relative increase of activated cTfh subsets frequencies at 1 year post-transplantation. In multivariate survival analysis, we reported that a decrease in activated CXCR5^+^PD1^+^ICOS^+^ at 1 year after transplantation in the blood of DSA-free patients was significantly associated with the risk of developing *de novo* DSA after the first year (*p* = 0.018, HR = 0.39), independently of HLA mismatches (*p* = 0.003, HR = 3.79). These results highlight the importance of monitoring activated Tfhs in patients early after transplantation and show that current treatments cannot provide early, efficient prevention of Tfh activation and migration. These findings indicate the need to develop innovative treatments to specifically target Tfhs to prevent DSA appearance in renal transplantation.

## Introduction

Both preexisting and *de novo* donor-specific anti-HLA antibodies (DSAs) are associated with chronic antibody-mediated rejection (ABMR), a leading cause of renal allograft loss (1–6). However, biological events associated with the appearance of DSAs are poorly characterized, and strategic options to block these events have limited efficacy because they are non-specific, administered too late or inefficient at targeting the source of antibodies (7, 8). A better understanding of the mechanisms leading to the formation, development, persistence, and action of DSAs is needed to guide the development of novel strategies to control DSAs and improve transplantation outcomes.

As a trigger of B cell antibody production, T follicular helper cells (Tfhs) are instrumental in promoting DSA appearance (9, 10). Tfhs are essential in the formation of germinal centers and development of an immunological memory response (11, 12). After interacting with B cells, Tfhs emerge from the germinal centers to become circulating Tfhs (cTfhs) (13, 14). Because of limited access to secondary lymphoid organs in humans, the existence of these cTfhs, which share the same functional capacities of germinal center, offers interesting opportunities for study and access. cTfhs can induce *in vitro* B lymphocyte differentiation (13, 14) and migrate into grafts where they can contribute to tertiary lymphoid organs that are associated with rejection (10, 15). Different subsets of cTfhs have been described with specific functions and phenotypes, with differential expression markers including CXCR3, inducible T cell costimulator (ICOS) and programmed cell death protein 1 (PD1) (16). Notably, PD1 has been associated as an activation marker of cTfhs and ICOS was found to be needed for cTfh homing and functions into germinal centers (14, 17). The expression of ICOS was also associated with expression of Ki67 showing cTfhs in active cell cycle (18). The use of CXCR3 distinguished cTfhs able to promote naïve B cell differentiation and immunoglobulins production, named Th2 (CXCR3^−^CCR6^−^) and Th17 (CXCR3^−^CCR6^+^), from CXCR3^+^CCR6^−^ Th1 cells unable to help naïve B cells (13). The link between cTFhs and antibody production has been clearly demonstrated; CXCR5^+^PD1^+^CXCR3^−^ cTfhs are correlated with anti-HIV antibody development (14), whereas CXCR5^+^CXCR3^+^ICOS^+^ cTfhs are associated with the development of antibodies after seasonal influenza vaccination by providing help to memory B cells (18). Based on these findings, these cTfh subsets with an activated phenotype may be instrumental in antibody production after renal transplantation.

Several studies in animal models suggest a beneficial effect of blocking the differentiation of cTfhs in transplantation (9, 19–23). In Humans, recent reports have shown an association of Tfhs with anti-HLA antibodies and/or DSA (10, 24–29). Interestingly, in accordance with these data, we recently reported reduced proportions of activated CXCR5^+^PD1^+^, CXCR5^+^PD1^+^ICOS^+^ and highly functional CXCR5^+^PD1^+^CXCR3^−^ cTfh subsets in blood from tolerant patients who stopped all immunosuppressive treatment while maintaining a functioning graft (25). This cTfh defect was linked to a low incidence of postgraft *de novo* donor-specific antibody (dnDSA) immunization (25). These data suggested that cTfhs may be present long before DSA appearance but have not being evidenced yet. Moreover, cTfhs can migrate and their presence in the graft is clearly associated with graft dysfunction, as attested in biopsies from patients experiencing acute T cell-mediated rejection, ABMR with anti-HLA-II DSAs and with mixed T and B cell-mediated rejection (10, 15, 29).

Examining their presence/clearance in blood from transplanted patients, particularly activated cTfh subsets linked to antibody production, may thus predict the genesis of antibody-mediated rejection as suggested in mouse model (23). Thus, herein, we measured the frequency of total CD4^+^CD45RA^−^CXCR5^+^ cTfhs and activated CXCR5^+^PD1^+^, CXCR5^+^PD1^+^ICOS^+^ and CXCR5^+^PD1^+^CXCR3^−^ subsets at the time of transplantation (day 0) and after 1 year (M12) in peripheral blood mononuclear cells (PBMCs) from a prospective cohort of 237 patients without DSA before renal transplantation and without any occurrence of dnDSA during the first year post-transplantation. By measuring cTfh levels before dnDSA appearance, in a large and prospective cohort of transplanted patients with a median follow-up of more than 7 years, we reported a significant association between the activated CXCR5^+^PD1^+^ICOS^+^ cTfh subset from DSA-free patients at M12 after transplantation and the appearance of dnDSA immunization after the first year post-transplantation. We also showed that depleting induction treatment was associated with a relative increase of activated cTfhs suggesting that current treatments did not provide efficient prevention of Tfh activation.

## Patients and Methods

### Study Population

This non-interventional research project involved data from the DIVAT cohort (www.divat.fr) and samples from the CENTAURE biocollection (www.fondation-centaure.org/) declared since 13/08/2008 to the Ministry of Research (N° PFS08-017) and hosted by the Center de Ressource Biologique (CRB) of Nantes University Hospital. Samples recorded in CRB software are in connection with the clinical data of the DIVAT database, in line with the good practice recommendations of the University Hospital of Nantes [approved by the CNIL (DR-2025-087 N° 914184, 15/02/2015) and the French Ministry of Higher Education and Research (file 13.334-cohort DIVAT RC12_0452)]. For each patient, written consent was obtained to use the clinical and laboratory data. The clinical and research activities being reported are consistent with the Principles of the Declaration of Istanbul. A total of 237 patients met the following inclusion criteria: adults, kidney or combined kidney and pancreas transplantation performed between March 2004 and December 2011, ABO-compatible transplantation, tissue from beating heart or deceased donors, no previous non-renal transplantation and no detectable DSA at day 0 and M12.

### Anti-HLA Immunization

Anti-HLA immunization was screened using a Luminex platform on a Labscan 100 flow analyzer (Luminex, Austin, TX, USA) with a multiantigen bead kits assay (LABSCreen Mixed, One Lambda Inc., Canoga Park, CA, USA) according to the manufacturer's instructions. Sera were collected at day 0, at M12 and during patient follow-up according to the standard of care. The cutoff value for a positive assay is above 2.5 normalized background ratio based on repeated laboratory testing. For positive screening, anti-HLA antibody specificities were defined using single-antigen beads coated with purified recombinant HLA class I and class II antigen (LABScreen Single Antigen; One Lambda Inc.). HLA A, B, C, DR, DQ, and DP antibodies with a normalized mean fluorescence intensity (MFI) > 1,000 were considered positive. Donor HLA typing was performed by PCR SSP (Olerup, Stockholm, Sweden), whereas patient HLA typing was performed by PCR SSO on a Luminex platform (One Lambda Inc.) or by Sanger Sequencing (Abbott, Chicago, IL, USA).

### cTfh Immunophenotyping

cTfh immunophenotyping was performed on frozen PBMCs as previously described (25) with a focus on the percentages of total cTfhs (CD4^+^CD45RA^−^CXCR5^+^), activated PD1^+^CD4^+^CD45RA^−^CXCR5^+^,PD1^+^ICOS^+^CD4^+^CD45RA^−^CXCR5^+^ cTfhs,PD1^+^CXCR3^−^CD4^+^CD45RA^−^CXCR5^+^ cTfhs (13, 14, 30). The following antibodies were used: anti-CD4 PerCP-Cy5.5 (L200), anti-CXCR5 Alexa 488 (RF8B2), anti-CXCR3 BV421 (1C6/CXCR3), anti-ICOS PE-Cy7 (C398.4A) (BD Bioscience, San Jose, CA, USA), anti-CD45RA APC (HI100; Miltenyi Biotec GmbH, Bergisch-Gladbach, Germany), and anti-PD1 PE (ebio-J105; eBiosciences, Vienna, Austria). Naïve (CD19^+^CD27^−^), memory (CD19^+^CD27^+^) and transitional (CD19^+^CD24^high^CD38^high^) B cells were analyzed using the following antibodies: anti-CD19 BUV395 (SJ25C1), anti-CD38 APCH7 (HB7), anti-CD24 PE-CF594 (ML5) (BD Biosciences), and anti-CD27 viobright-FITC (MT271) (Miltenyi Biotec GmbH).

Dead cells were excluded by a LIVE/DEAD fixable yellow dye (Invitrogen, Carlsbad, CA, USA). Cells were analyzed on an LSRII flow cytometer (BD Biosciences). Analyses of blood cTfhs and CD19^+^ B lymphocytes were performed blindly using FlowJo v.9.7.6 (TreeStar Inc., Ashland, OR, USA) with a standard gating strategy using Fluorescence Minus One (FMO) controls as displayed in Supplementary Figure 1. Absolute numbers were calculated using lymphocyte counts obtained from hemograms performed at the hospital and the cTfh frequencies we measured.

### Statistical Analysis

The main judgment criterion was the time from transplantation to the first occurrence of dnDSA or the last follow-up. Kaplan-Meier survival curves and the log-rank test were used to study the impact of variables on the appearance of dnDSA. Cox regression multivariate analysis was performed including the following predefined clinically relevant variables: recipient age, transplantation rank (one for a first transplantation, two or more in case of re-transplantation), immune event and other variables potentially associated with DSA in univariate analysis with a *p*-value inferior to 0.20 in order not to bias parameters selection (31). A reduced model containing only significant (*p* < 0.05) and independent variables associated with DSA appearance was obtained using a backward procedure, and hazard ratios (HRs) with 95% confidence intervals (95% CIs) were calculated. As the cTfh variable distribution did not fit the log-linearity hypothesis, the results are given for discretized parameters according to the median. Analyses were performed using R v.3.4.3. Multiple group comparisons were performed using Kruskal-Wallis with Dunn's *ad hoc* pairwise comparisons, and Mann-Whitney or paired Wilcoxon tests were used for comparisons of two groups using GraphPad Prism v.6 (GraphPad Software, La Jolla, CA, USA).

## Results

### Demographic Description of the Cohort of Transplanted Patients

**Figure 1:**
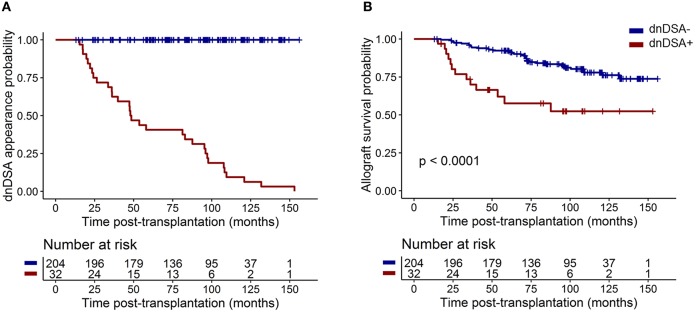
dnDSA appearance in the 237 patients from the cohort. **(A)** Cumulative probability of dnDSA during patient follow-up. **(B)** Allograft survival (including recipient death) for patients exhibiting dnDSA after M12 was worse than that for patients who did not develop dnDSA during follow-up (log-rank test *p* < 0.0001).

Table 1 summarizes the clinical characteristics of the 237 transplanted patients. All patients were alive with a functional graft at M12 and received standard maintenance immunosuppressive therapy with calcineurin inhibitors (CNIs: 97%; mainly Tacrolimus: 86.5%), antiproliferative agents (98.7%; mycophenolic acid (MPA) or azathioprine), and a corticosteroid regimen (74.3%). Eighty-seven patients (36.7%) were treated with anti-thymocyte globulin (ATG)-depleting induction treatment, and 150 (63.3%) were treated with basiliximab non-depleting treatment (*n* = 145) or received no induction therapy (*n* = 5). All patients were DSA free at day 0 and M12. Thirty-two patients (13.60%) developed dnDSA after M12 with a median time of dnDSA appearance of 48.05 months [interquartile range (IQR) = 24.6–96.6; Figure 1A]. Patients who developed dnDSA over 1 year after transplantation exhibited worse allograft survival than did those without dnDSA, with an overall median follow-up of 89.61 months after transplantation (IQR = 62.8–108.1; Figure 1B, *p* < 0.0001).

**Table 1:** Patient characteristics.

**Variables**	**Mean [IQR]**	**Missing values**	**Log-rank test *p*-value**
Recipient age	48.0 [39–57]	0	0.38
Time post-transplantation to develop dnDSA	48.05 [24.6–96.6]	0	ND
Donor age	47.35 [38–58.5]	0	0.78
**Variables**	**Number (%)**	**Missing values**	**Log-rank test** ***p*****-value**
**Recipient gender**			
Male	152 (64.14)	0	0.46
Female	85 (35.86)		
**Initial disease**			
Undetermined etiology	19 (8.02)	0	0.63
Chronic glomerulonephritis	58 (24.47)		
Chronic interstitial nephritis, urinary, and others malformations	96 (40.50)		
Vascular renal diseases	16 (6.75)		
Diabetes	48 (20.25)		
**Renal replacement therapy before transplantation**			
Preemptive transplantation	44 (18.57)	0	0.77
Peritoneal dialysis	24 (10.13)		
Hemodialysis	169 (71.31)		
**Allograft**			
Kidney	200 (84.39)	0	0.25
Pancreas-Kidney	37 (15.61)		
**Allograft rank**			
First	216 (91.14)	0	0.52
Second	19 (8.02)		
Third	2 (0.84)		
**Donor type**			
Deceased	216 (91.14)	0	0.40
Live	21 (8.86)		
**Deceased donor type**			
Non-beating heart	3 (1.39)	21	0.18
Beating heart	213 (98.61)		
**HLA mismatches**			
<4	112 (47.26)	0	**0.0018**
≥4	125 (52.74)1		
**Induction treatment**			
Non-depleting or none	150 (63.29)	0	**0.11**
Depleting	87 (36.71)		
**Immunization anti-HLA class I at day 0**			
Negative	220 (92.83)	0	0.16
Positive	17 (7.17)		
**Immunization anti-HLA class II at day 0**			
Negative	210 (88.61)	0	0.48
Positive	27 (11.39)		
**Previous immunizing event (blood transfusion/pregnancy)**			
Yes	107 (45.15)	0	0.30
No	130 (54.85)		
**Immunosuppression**			
Cyclosporine A	24 (10.13)	2	0.23 (Tacrolimus vs. Cyclosporine A)
Tacrolimus	205 (86.50)		
Belatacept	4 (1.69)		
mTOR inhibitor	2 (0.84)		
**Corticosteroid**			
Yes	176 (74.26)	0	0.79
No	61 (25.74%)		
Delay graft function	75 (31.65)	0	0.59
**Rejection during the first year**			
0	216 (91.14)	0	**0.059**
1	17 (7.17)		
2	4 (1.69)		
**Reject type**			
Borderline	1 (4.76)	0	0.63
Cellular	17 (80.95)		
Mixed	3 (14.29)		

ND, Not determined; IQR, Interquartile range.

Since no patient with anti-HLA class I at day 0 displayed dnDSA, this parameter was not used in the multivariate Cox model.

*The log-rank test was used to study the impact of variables on dnDSA appearance. Variables with a p-value for the univariate Cox regression <0.2 (in bold) were included in multivariate Cox regression analyses*.

Continuous variables are described using means and interquartile ranges (IQR), whereas categorical variables are described using frequencies and percentages.

### cTfh Levels Are Affected by Hemodialysis and Immunizing Events Before Transplantation

**Figure 2:**
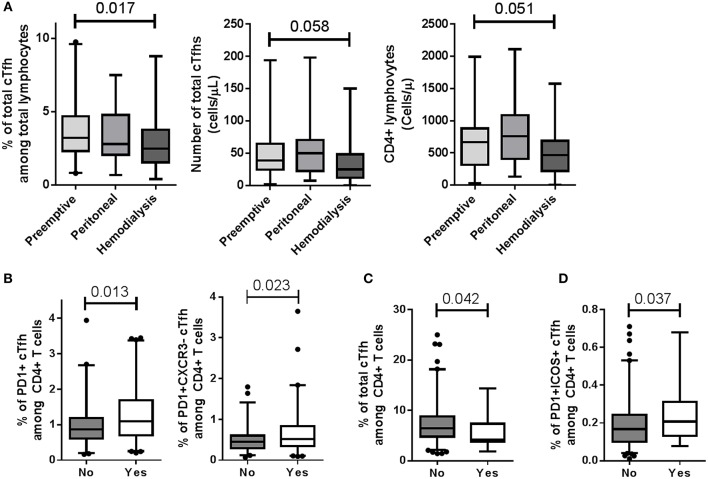
Modulation of cTfh levels at day 0. **(A)** Hemodialysis significantly decreased total cTfh frequency among total lymphocytes compared to pre-emptive transplantation (3.21 vs. 2.48%; *p* = 0.017; Kruskal-Wallis with Dunn's pairwise comparisons). Trends to decrease of CD4^+^ lymphocyte and cTfh counts were also observed in patients receiving a pre-emptive transplantation (*p* = 0.051 and 0.058, respectively). **(B)** Patients who experienced an immunizing event (Yes), *i.e*., previous blood transfusion and/or pregnancy, displayed an increased frequency of CXCR5^+^PD1^+^ and CXCR5^+^PD1^+^CXCR3^−^ cTfh populations (left, *p* = 0.013 and right, 0.023, respectively), compared to those who did not (No). **(C)** The presence of class I anti-HLA antibodies (non-DSA) (Yes) was associated with a decrease of total cTfhs (*p* = 0.042). **(D)** The percentage of CXCR5^+^PD1^+^ICOS^+^ cTfh subsets was higher in patients with class II non-DSA anti-HLA antibodies (Yes) than in patients with no anti-HLA antibody (No) (0.17 vs. 0.21%, *p* = 0.037). Whisker boxes with 95% confidence intervals are displayed. *P*-values of Mann-Whitney tests are indicated.

We assessed whether recipient characteristics at day 0 were associated with total CD4^+^CD45RA^−^CXCR5^+^ cTfhs and/or activated PD1^+^, PD1^+^ICOS^+^ cTfh and PD1^+^CXCR3^−^ cTfh subsets. No association was found between total cTfhs and cTfh subsets with the initial disease, previous transplantation, diabetes, recipient age at transplantation, and recipient gender (data not shown). The total cTfh level within total lymphocytes at day 0 was lower in patients who received hemodialysis than in those who received a pre-emptive transplantation (3.21 vs. 2.48%; *p* = 0.017) which was associated with trends to decrease of CD4^+^ lymphocytes and cTfh cell numbers in patients receiving pre-emptive transplantation (*p* = 0.051 and 0.058, respectively; Figure 2A and Supplementary Figure 2). The percentages of activated CXCR5^+^PD1^+^ and CXCR5^+^PD1^+^CXCR3^−^ cTfh populations in CD4^+^ lymphocytes were significantly increased at day 0 (*p* = 0.013 and 0.023, respectively) in patients who experienced previous immunizing events, including previous blood transfusions and/or pregnancies (Figure 2B). No difference in their absolute numbers were evidence (data not shown). While the presence of class I anti-HLA antibodies (non-DSA) was associated with a decrease of total cTfh (*p* = 0.042, Figure 2C), the presence of class II anti-HLA antibodies (non-DSA) was associated with a significant increase in activated CXCR5^+^PD1^+^ICOS^+^ cTfh subsets (0.17 vs. 0.21%, *p* = 0.037; Figure 2D) with no modification of their absolute numbers.

### One Year Post-transplantation, cTfhs Are Affected by Induction and Maintenance Treatments

**Figure 3:**
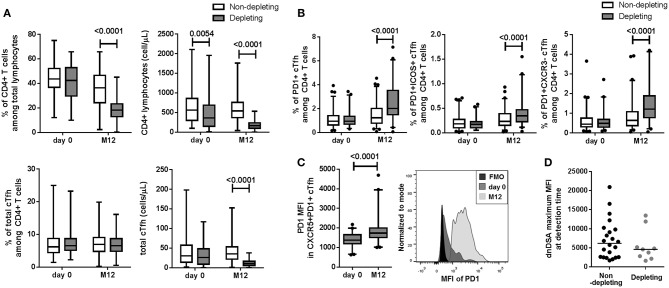
Depleting induction treatment altered cTfh frequency at M12. **(A)** Compared to non-depleting or no induction treatment (white boxes), the ATG-depleting induction treatment (gray boxes) was associated with a decrease in CD4^+^ T lymphocyte frequency and number, a decrease in total cTfh number at M12 (*p* < 0.0001) but not with total cTfh frequency. **(B)** Increase frequencies of the three CXCR5^+^PD1^+^, CXCR5^+^PD1^+^ICOS^+^, and CXCR5^+^PD1^+^CXCR3^−^ activated cTfh subsets were associated with depleting treatment at M12 (**B**, *p* < 0.0001). (**C**, left) Depleting treatment was associated with an increase of PD1 MFI in CXCR5^+^PD1^+^ cTfhs at M12 compared to day 0 (*p* < 0.0001). (**C**, right) Representative histograms of PD1 MFI in total cTfhs from one patient at day 0 and M12 are displayed. **(D)** No difference between dnDSA intensity (maximum of MFI) at detection time was observed (*p* = 0.41). For **A**, **B**, and **C**, whisker boxes with 95% confidence intervals are displayed. *P*-values of paired Wilcoxon (for **A**, **B**, and **C**) and Mann-Whitney **(D)** tests are indicated.

We analyzed the effect of the ATG depleting induction treatment compared to basiliximab non-depleting treatment or the absence of induction therapy on the frequency of total CD4^+^CD45RA^−^CXCR5^+^ cTfhs and activated CXCR5^+^PD1^+^, CXCR5^+^PD1^+^ICOS^+^ and CXCR5^+^PD1^+^CXCR3^−^ cTfh subsets at M12. Unsurprisingly, the percentage of CD4^+^ T lymphocytes from total lymphocytes and their absolute number were decreased at M12 and was accompanied with a decrease total cTfh number in patients receiving ATG depleting induction treatment (Figure 3A, *p* < 0.0001). While no difference in the frequency of total cTfhs from CD4^+^ lymphocytes was observed (Figure 3A), depleting treatment was associated with a significant increase in the frequency of the three CXCR5^+^PD1^+^, CXCR5^+^PD1^+^ICOS^+^, and CXCR5^+^PD1^+^CXCR3^−^ activated cTfh subsets at M12 and no difference was observed at day 0 (Figure 3B, *p* < 0.0001). The expression levels of PD1 (MFI) in activated CXCR5^+^PD1^+^, cTfhs was significantly higher in patients treated with ATG at M12 than at day 0 (Figure 3C, *p* < 0.0001). In addition, there was no difference in dnDSA intensity (maximum MFI) measured at detection time between patients who received a depleting treatment and those who received other treatments (*p* = 0.41, Figure 3D).

**Figure 4:**
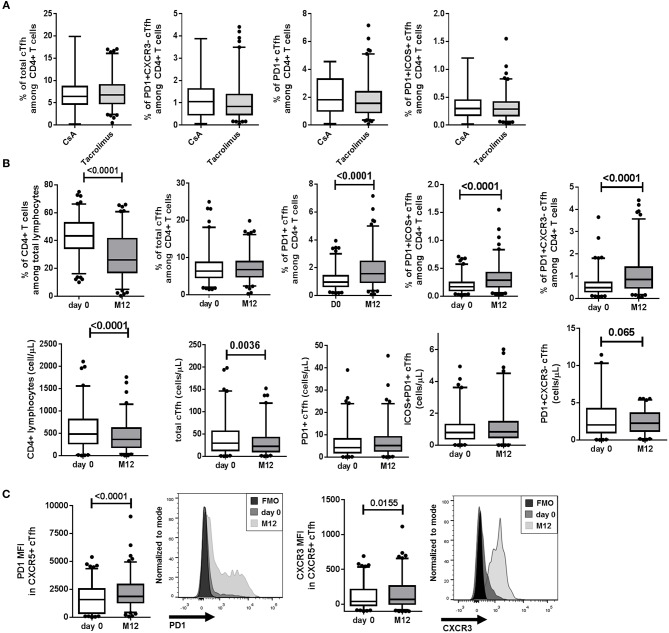
CNI treatment altered cTfh frequency at M12. **(A)** At M12, no difference within cTfh populations was observed between patients treated with CsA and Tacrolimus (white and gray boxes, respectively). **(B)** Percentages (top) and absolute cell numbers (bottom) are displayed for CD4^+^ lymphocytes, total CD4^+^CD45RA^−^CXCR5^+^ cTfhs and the three CXCR5^+^PD1^+^, CXCR5^+^PD1^+^ICOS^+^, and CXCR5^+^PD1^+^CXCR3^−^ activated cTfh subsets comparing day 0 and M12 for the 229 CNI-treated patients (CsA and Tacrolimus). **(C)** The expression of PD1 and CXCR3 (MFI) in total cTfhs are showed aside with representative histograms from one patient at day 0 and M12 (*p* < 0.0001 and 0.0155, respectively). Whisker boxes with 95% confidence intervals are displayed. *P*-values of Mann-Whitney **(A)** and paired Wilcoxon (for **B** and **C**) tests are indicated.

A majority of patients received maintenance CNI therapy (97%), including Tacrolimus (86.5%, *n* = 205) or Cyclosporin A (CsA, 10.13%, *n* = 24). No difference within cTfh populations was observed between patients treated with CsA and Tacrolimus (Figure 4A). Irrespective of the type of treatment (CsA or Tacrolimus), and similarly to the effect of ATG depleting induction treatment, the percentage of CD4^+^ lymphocytes from total lymphocytes and their absolute numbers were decreased comparing day 0 and M12 for CNI-treated patients (Figure 4B, *p* < 0.0001). Comparing day 0 and M12 for CNI-treated patients, we found no significant decrease level of total CD4^+^CD45RA^−^CXCR5^+^ cTfhs from total CD4^+^ lymphocytes but relative increase frequencies of the three CXCR5^+^PD1^+^, CXCR5^+^PD1^+^ICOS^+^, and CXCR5^+^PD1^+^CXCR3^−^ activated cTfh subsets at M12 (*p* < 0.0001, Figure 4B). Regarding cell numbers, according to the clear decrease of total CD4^+^ T lymphocytes, a decrease of total cTfh was observed whereas absolute numbers of CXCR5^+^PD1^+^, CXCR5^+^PD1^+^ICOS^+^, and CXCR5^+^PD1^+^CXCR3^−^ activated cTfh subsets were not different (Figure 4B). The expression of PD1 and CXCR3 (MFI) in total cTfhs was significantly higher at M12 than at day 0 (Figure 4C, *p* < 0.0001 and 0.0155, respectively). Finally, no analyzed cell population was found to be significantly associated with corticoids administration among patients not receiving ATG depleting induction treatment (*p* > 0.05). Altogether, induction and immunosuppressive maintenance treatments were associated with a decreased number of total cTfhs and a relative increase in activated cTfh subsets at M12.

### HLA Mismatches and 1 Year CXCR5^+^PD1^+^ICOS^+^ cTfh Frequency Are Significantly and Independently Associated With dnDSA Appearance

We tested the clinical variables and percentages of total CD4^+^CD45RA^−^CXCR5^+^ cTfhs and activated CXCR5^+^PD1^+^, CXCR5^+^PD1^+^ICOS^+^, and CXCR5^+^PD1^+^CXCR3^−^ cTfh subsets from CD4^+^ lymphocytes in univariate survival analysis for an association with dnDSA appearance (Table 1). Only these four cTfh populations were included in a hypothesis-driven study based on previous results (25) to reduce statistical overfitting. Three clinical variables were associated with dnDSA appearance after 1 year, including HLA mismatches (A-B-DR ≥ 4; *p* = 0.0018), acute rejection during the first year post-transplantation (*p* = 0.059) and non-depleting induction treatment (*p* = 0.11). We found no association between the percentages of total cTfhs and subsets at day 0 or variations between day 0 and M12 and dnDSA appearance. Interestingly, the percentage of total CD4^+^CD45RA^−^CXCR5^+^ cTfhs (*p* = 0.096; HR = 0.53, 95% IC = [0.26–1.11]) and activated CXCR5^+^PD1^+^ICOS^+^ cTfh (*p* = 0.015; HR = 0.39, 95% IC = [0.18–0.86]) and CXCR5^+^PD1^+^CXCR3^−^ subsets (*p* = 0.14; HR = 0.58, 95% IC = [0.28–1.22]) at M12 in DSA-free patients were associated with dnDSA appearance after 1 year [with *p* < 0.20 for univariate analysis (31)]. In addition, both at day 0 and M12, frequencies of naïve, memory and transitional B cells were not associated with dnDSA in this univariate analysis (*p* > 0.20).

**Figure 5:**
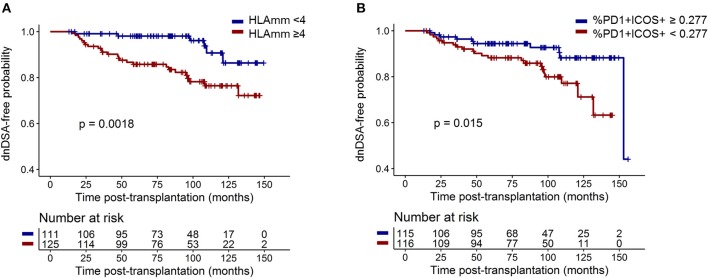
HLA mismatches and M12 CXCR5^+^PD1^+^ICOS^+^ cTfh frequency were significantly and independently associated with dnDSA appearance. Survival curves of dnDSA appearance for **(A)** HLA mismatches (threshold of 4 mismatches) and **(B)** M12 CXCR5^+^PD1^+^ICOS^+^ cTfh frequency among CD4^+^ T cells (threshold is the median frequency: 0.277%) are displayed. *p*-values of the log-rank test are displayed.

Next, the six significant clinical and biological parameters (HLA mismatches; acute rejection and depleting induction treatment; and CD4^+^CD45RA^−^CXCR5^+^, CXCR5^+^PD1^+^ICOS^+^, and CXCR5^+^PD1^+^CXCR3^−^ cTfhs) were tested in multivariate survival analysis for an association with dnDSA appearance. Despite being non-significant, age at transplantation (*p* = 0.38), transplantation rank (*p* = 0.52), and immunizing events before transplantation, i.e., blood transfusion and/or pregnancy (*p* = 0.30), were intentionally included in the analysis as classical risk factors of dnDSA (6). Two parameters were independently associated with dnDSA appearance after M12. Specifically, patients with four or more HLA mismatches had a 3.79 higher risk of developing dnDSA (*p* = 0.003; HR = 3.79; 95% IC = [1.56–9.25]) than did those with fewer HLA mismatches, and DSA-free patients with more than 0.277% CXCR5^+^ PD1^+^ ICOS^+^ cTfhs in their blood at M12 had a lower risk of developing dnDSA after M12 [*p* = 0.018; HR = 0.39, 95% IC = [0.18–0.85]; Table 2, Figure 5]. These data demonstrated that HLA mismatches and M12 CXCR5^+^PD1^+^ICOS^+^ cTfh levels were significantly and independently associated with dnDSA appearance after M12 in our cohort.

**Table 2:** CXCR5^+^PD1^+^ICOS^+^ cTfhs and HLA mismatches were significantly and independently associated with DSA.

**Variables**	**Multivariate analysis**		**Reduced model**	
	**Adjusted HR [95% CI]**	***p*-value**	**Adjusted HR [95% CI]**	***p*-value**
M12 total cTfh % Median threshold (1.6.76%)	0.72 [0.29–1.80]	0.48	–	–
**M12 CXCR5**^**+**^**PD1**^**+**^**ICOS**^**+**^ **cTfh** **Median threshold (0.277%)**	**0.37 [0.14–1.00]**	**0.051**	**0.39 [0.18–0.85]**	**0. 018**
CXCR5^+^PD1^+^CXCR3^−^ cTfh Median threshold (0.8%)	1.46 [0.47–4.54]	0.51	–	–
Recipient Age (>55 years)	1.28 [0.59–2.79]	0.53	–	–
Allograft rank (>1)	1.80 [0.34–9.51]	0.49	–	–
Previous immunizing event	2.03 [0.96–4.31]	0.064	–	–
**HLA mismatches (A, B, and DR ≥4)**	**4.36 [1.73–11.0]**	**0.0018**	**3.79 [1.56–9.25]**	**0.003**
Depleting induction treatment	0.47 [0.17**–**1.29]	0.14	–	–
Rejection during the first year	2.26 [0.77–6.66]	0.14	–	–

*Recipient age, transplantation rank, immune events, and other variables potentially associated with DSA in the univariate analysis p ≤ 0.20 in the univariate Cox regression analysis (31) were included in a complete model of multivariate Cox survival analysis. The reduced model was built with a backward procedure and parameters with a p value < 0.05 in the reduced model were highlighted in bold. Continuous parameters were dichotomized according to the median to fulfill log-linearity assumptions*.

## Discussion

The occurrence of dnDSA after transplantation is associated with an increase in the prevalence of ABMR and the risk of graft loss (1–6). Thus, monitoring dnDSA after transplantation is instrumental and recognized as a standard of care for renal transplant recipients. Tfhs are the main cells orchestrating DSA production by aiding B cells through different molecules, such as ICOS (32), CD40L and PD1, as well as various soluble factors (Il-4, Il-6, and Il-21) (12, 13, 33, 34). Tfhs are composed of phenotypically and functionally different subsets based on the expression of these molecules and their role has been demonstrated in several pathologies in humans (14, 18, 35).

Blood cTfhs share similar functional properties with classical Tfhs from the germinal center (13). In transplantation, cTfhs are associated with renal transplant outcomes (10, 24–27, 29). Specifically, higher levels of cTfhs (CD4^+^CXCR5^+^) were observed at diagnosis in patients who experienced ABMR (24), ABMR with anti-HLA class II DSA (29), and in patients with preexisting DSA at 3 months post-transplantation (10). A higher absolute number of cTfhs before transplantation was associated with acute rejection experiences during the first year of transplantation (27). Interestingly, in a cohort of tolerant patients, we observed a decrease in activated cTfh subsets (CXCR5^+^PD1^+^, CXCR5^+^PD1^+^ICOS^+^, and CXCR5^+^PD1^+^CXCR3^−^) without affecting the absolute number of cTfhs (25). In these previous studies, cTh levels were measured at or after diagnostics, for chronic rejection (10, 24, 29) or tolerance (25). However, no study has yet demonstrated cTfh levels as predictive of DSA appearance in the long term in Human. To answer this point, we measured cTfhs in a large and prospective cohort of 237 DSA-free patients at day 0 and M12 with a follow-up of 89.6 months after transplantation. We reported that the M12 CXCR5^+^PD1^+^ICOS^+^ cTfh level was significantly and independently associated with dnDSA appearance after 1 year post-transplantation. DSA-free patients with lower levels of CXCR5^+^PD1^+^ICOS^+^ cTfhs at M12 had a higher risk of developing dnDSA after the first year post-transplantation when B cell modifications could not be detected. Furthermore, in our prospective study, patients who developed dnDSA over 1 year after transplantation exhibited worse allograft survival than patients who did not develop dnDSA (*p* < 0.0001), in accordance with other reports evidencing dnDSA appearance was a negative risk factor to allograft survival (1–6).

Cano-Romero's group recently reported that patients who develop *de novo* anti-HLA antibodies during the first year post-transplantation display an increased number of PD1^+^ cTfhs up to 6 months after transplantation (27). The authors analyzed *de novo* anti-HLA antibody appearance during the first year post-transplantation, whereas we focused on *dn*DSA occurrence after the first year of transplantation in patients who were DSA-free in this first year. In a murine model of skin transplantation, a rapid increase of donor-specific CXCR5^+^PD1^+^ICOS^+^ cTfhs in blood preceded the appearance of DSA, followed by a contraction phase (23). By contrast, we did not observe an increase of these cTfhs but a decrease level associated with dnDSA which may be explained by different kinetics between mice and human (days compared to months or years). Our different endpoints and results suggest different cell kinetics for cTfhs, particularly activated CXCR5^+^PD1^+^ICOS^+^ cTfhs, which may have expanded the first months after transplantation according Cano-Romero et al. results, and then migrated later after transplantation in the graft and decreased in peripheral blood (27). In line with this hypothesis, Shi et al. evidenced an increase of CD4^+^CXCR5^+^ cTfhs in peripheral blood at diagnosis of ABMR with a decrease expression of PD1 that we could linked to the decrease frequency of activated cTfhs we observed (24). Indeed, ICOS is essential to the localization of CD4^+^ T cells within follicles and to support a germinal center response (17) and cTfhs have been evidenced to migrate toward inflamed organs such as in lungs infected with influenza virus (36). We hypothesized the decreased frequency of activated cTfhs suggests their migration toward draining lymph nodes to support antibody production (19) or migration into the graft where local inflammation is taking place to participate in the formation of tertiary organs and establishing a local memory compartment (10, 16, 29, 37). Indeed, according literature, cTfhs are likely to be present at diagnosis in biopsies from patients experiencing allograft rejection (10, 15, 20, 29). Liarski et al. demonstrated that CD4^+^PD1^+^ICOS^+^ cells were found in 50% of renal biopsies with mixed T and B cell-mediated rejection and expressed high levels of *IL21* mRNA, which is a key cytokine to promote B cell maturation (15). The presence of CD3^+^CXCR5^+^ Tfhs have been evidenced in biopsies from patients exhibiting anti-class II DSA and ABMR as well as the presence of Bcl-6-expressing Tfhs on the T-B cell border of the follicular-like structures in biopsies from acute TCMR (10, 29). We also evidenced the presence of CD4^+^PD1^+^ T cells co-expressing ICOS and IL-21 in a model of renal transplantation (20). However, our current analysis was focused on peripheral blood as a non-invasive compartment which also allows repeated measures for immune monitoring. Evidences of cTfh roles in dnDSA formation and allograft rejection would require further investigations, notably in a longitudinal study with blood and matched biopsies.

In our cohort, ATG treatment was associated with a reduction in the total CXCR5^+^ cTfh number at M12. ATG induces depletion of both CD4^+^ and CD8 T^+^ cells (38), and cTfh cells represent approximately 15% of total circulating CD4^+^ T cells in healthy individuals. Thus, unsurprisingly, induction treatment and standard immunosuppressive therapies decreased cTfh levels as previously reported (27, 28, 39). Our data are also consistent with results from Thaunat's group who reported prolonged cTfh depletion after ATG induction therapy and reduced CD40L and ICOS expression on activated cTfhs (28). In our cohort, this decreased frequency of total cTfhs was associated with increased frequencies of activated cTfh subsets (CXCR5^+^PD1^+^, CXCR5^+^PD1^+^ICOS^+^, and CXCR5^+^PD1^+^CXCR3^−^ cTfhs) in CNI-treated recipients and ATG induction therapy, as well as higher expression of cTfh activation marker PD1 at M12. The frequency of activated cTfh subsets is also increased by immunizing events, including previous blood transfusions and/or pregnancies, and the presence of class II non-DSA anti-HLA antibodies. These data, in accordance with previous reports, suggest that pre-sensibilization increases the pool of activated cTfhs (10, 27, 40) and is consistent with the lack of effect of standard immunosuppression on activated Tfhs (23, 28). These results are also in line with reports exhibiting that T cells were unequally targeted by immunosuppressive treatments, memory T cells exhibiting a relative increase frequency after ATG depletion (41). In addition, we were not able to identify significant difference in cTfh distribution between patients treated with corticoid treatment or not. These data clearly suggest that current immunosuppressive therapies cannot (at least not efficiently) target activated Tfhs, which are potentially harmful for the graft. T cells that are refractory to depletion likely continue to aid B cells. As a limitation of our study, cTfh phenotype could be altered by cell freezing, however, we did not observe any impact of storage time on viability percentages or total cTfh frequency (Supplementary Figure 3). Due to unbalanced administration of other immunosuppressive treatments, we could not analyze their effects on cTfh levels, limiting our ability to generalize our conclusion to all immunosuppressive treatments.

In conclusion, our study shows that one-year CXCR5^+^PD1^+^ICOS^+^ cTfh level was significantly and independently associated with dnDSA appearance after 1 year post-transplantation, before dnDSA appearance, and that current immunosuppressive therapies are unable to efficiently target the pool of activated cTfhs. DSA-free patients with lower levels of M12 CXCR5^+^PD1^+^ICOS^+^ cTfhs had a higher risk of developing dnDSA after the first year post-transplantation. Tfhs are involved in all types of rejection; beyond mixed or antibody-mediated effects alone, the maintenance of their function under immunosuppression allows B cells to exert their cytotoxic and antigen-presenting functions. Our results encourage the development of immunosuppressive strategies targeting Tfhs and activated subsets, specifically CXCR5^+^PD1^+^ICOS^+^ cTfhs, to prevent dnDSA appearance and the risk of CAMR. Clearly, these data also raise the need of following Tfh to predict DSA occurrence in patients.

## Data Availability

The raw data supporting the conclusions of this manuscript will be made available by the authors, without undue reservation, to any qualified researcher.

## Ethics Statement

This non-interventional research project involved data from the DIVAT cohort (www.divat.fr) and samples from the CENTAURE biocollection (www.fondation-centaure.org/) declared since 13/08/2008 to the Ministry of Research (N° PFS08-017) and hosted by the Centre de Ressource Biologique (CRB) of Nantes University Hospital. Samples recorded in CRB software are in connection with the clinical data of the DIVAT database, in line with the good practice recommendations of the University Hospital of Nantes [approved by the CNIL (DR-2025-087 N° 914184, 15/02/2015) and the French Ministry of Higher Education and Research (file 13.334-cohort DIVAT RC12_0452)]. For each patient, written consent was obtained to use the clinical and laboratory data. The clinical and research activities being reported are consistent with the Principles of the Declaration of Istanbul.

## Author Contributions

ACh, MG, and SB designed the study. MC, FD, and SL carried out the experiments. RD, MC, FD, CK, and ACe analyzed the data. RD, MC, SC, ND, MG, and SB drafted and revised the paper. All authors approved the final version of the manuscript.

### Conflict of Interest Statement

The authors declare conflicts of interest with regard to Roche Pharma, Novartis, Sanofi, and Astellas laboratories supporting the DIVAT cohort.

## Abbreviations

95% CI: 95% confidence interval
ATG: anti-thymocyte globulin
CIT: cold ischemia time
CsA: cyclosporine A
cTfh: circulating Tfh
day 0: transplantation time
dnDSA: *de novo* DSA
DSA: donor specific antibody
HLA: human leukocyte antigen
IQR: interquartile range
LB: lymphocyte B
LT: lymphocyte T
M12: 1 year post-transplant
MPA: mycophenolic acid
SD: standard deviation
Tfh: T follicular helper lymphocytes.
