# Supplementary material for: CXCR5+PD1+ICOS+ Circulating T Follicular Helpers Are Associated With de novo Donor-Specific Antibodies After Renal Transplantation
Source: Front Immunol. 2019 Sep 10;10:2071. doi: 10.3389/fimmu.2019.02071 (PMC6746839; doi:10.3389/fimmu.2019.02071)
Supplement: Supplementary file 1 [file Presentation_1.pdf]

SUPPLEMENTAL MATERIAL

Supplemental material table of content:

Supplementary Figure 1 p.S2

Supplementary Figure 2 p.S4

Supplementary Figure 3 p.S5

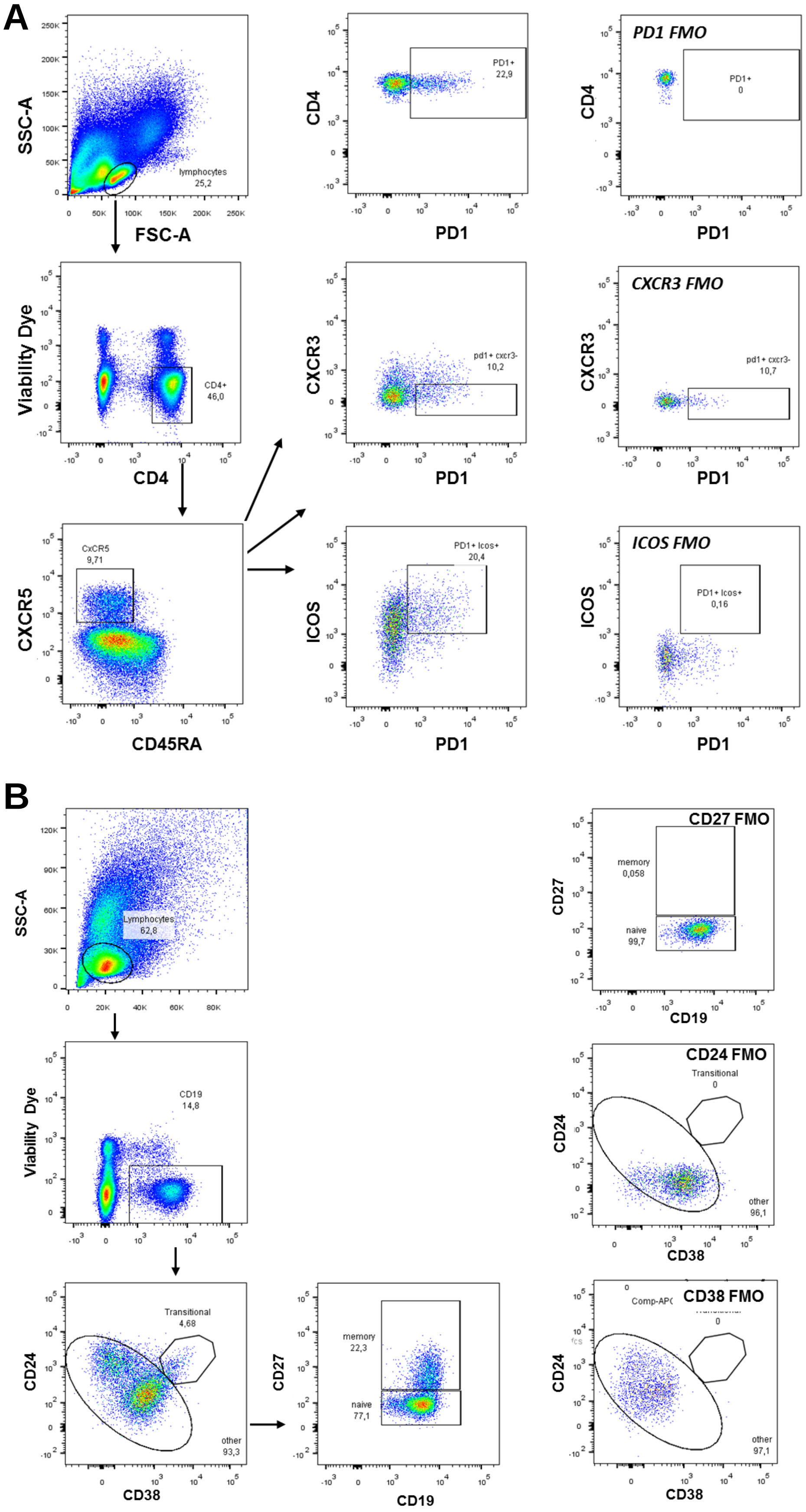
A
SSC-A
FSC-A
lymphocytes
25,2
Viability Dye
CD4
CD4+
46,0
CXCR5
CD45RA
CxCR5
9,71
PD1
PD1+
22,9
CXCR3
pd1+ cxcr3-
10,2
ICOS
PD1+ Icos+
20,4
PD1 FMO
PD1+
0
CXCR3 FMO
pd1+ cxcr3-
10,7
ICOS FMO
PD1+ Icos+
0,16
B
SSC-A
Lymphocytes
62,8
Viability Dye
CD19
14,8
CD24
CD38
Transitional
4,68
other
93,3
CD27
CD19
memory
22,3
naive
77,1
CD27 FMO
memory
0,058
naive
99,7
CD24 FMO
Transitional
0
other
96,1
CD38 FMO
0
other
97,1

Supplementary Figure 1: Representative dot plots illustrating the gating strategy of (A) total cTfhs ($CD4^{+}CD45RA^{-}CXCR5^{+}$), $CD4^{+}CD45RA^{-}CXCR5^{+}PD1^{+}$, $CD4^{+}CD45RA^{-}CXCR5^{+}PD1^{+}ICOS^{+}$ and $CD4^{+}CD45RA^{-}CXCR5^{+}PD1^{+}CXCR3^{-}$ cTfhs (left and middle panels); and (B) naïve ($CD19^{+}CD27^{-}$), memory ($CD19^{+}CD27^{+}$) and transitional ($CD19^{+}CD24^{high}CD38^{high}$) B cells. Fluorescence Minus One (FMO) controls were used to determine the optimal gate position as displayed on right panels.

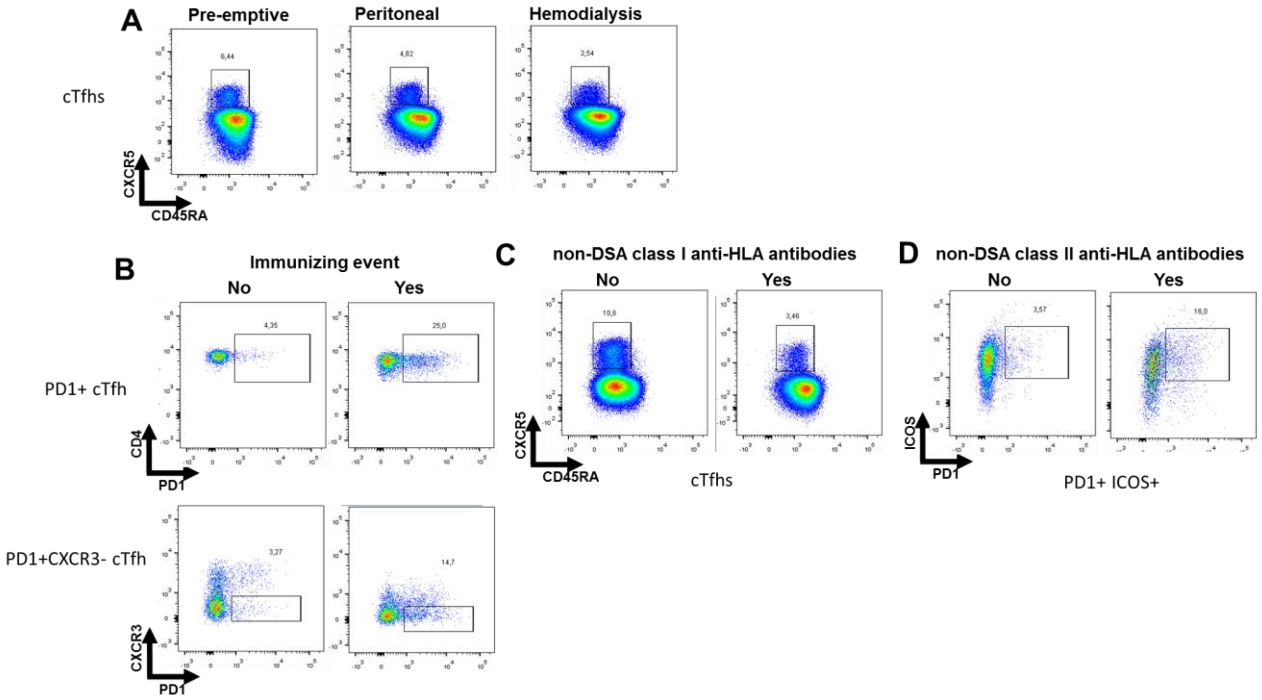

Supplementary Figure 2: Representative cytometry dot plots at transplantation. A) Representative dot plots of total cTfhs ($CD4^+CD45RA^-CXCR5^+$) at day 0 for patients receiving pre-emptive transplantation, peritoneal dialysis and hemodialysis). B) Representative dot plots of $CXCR5^+PD1^+$ (top) and $CXCR5^+PD1^+CXCR3^-$ (bottom) cTfhs in patients who experienced an immunizing event (Yes), compared to those who did not (No). C) Representative dot plots of total cTfh from $CD4^+$ lymphocytes for patients with class I anti-HLA antibodies (non DSA) (Yes) and those with none (No). D) Representative dot plots of $CXCR5^+PD1^+ICOS^+$ cTfh subset in patients with class II non-DSA anti-HLA antibodies (Yes) and in patients with no anti-HLA antibody (No).

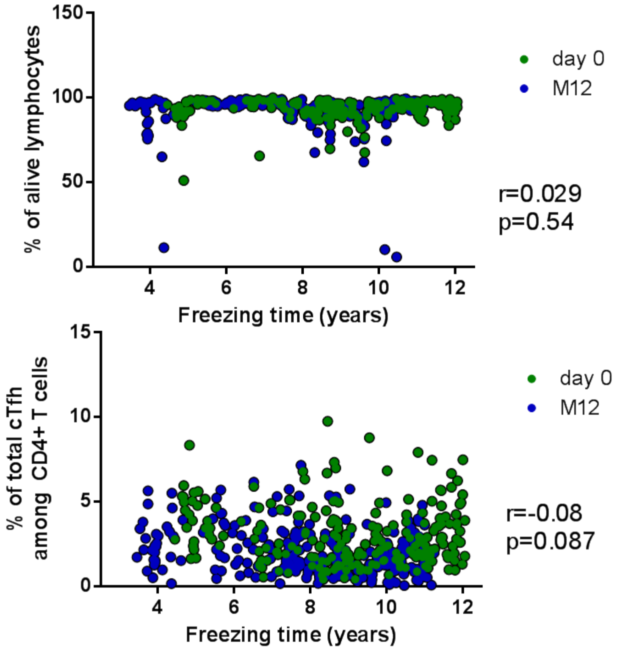

Supplementary Figure 3: No correlation was evidenced between freezing time (in years) and frequency of alive lymphocytes (upper panel) or total $CD4^{+}CD45RA^{-}CXCR5^{+}$ cTfh frequency (lower panel). P-values from Spearman correlation are indicated.
